# Higher Nitrogen Uptakes Contribute to Growth Advantage of Invasive *Solanum rostratum* over Two Co-Occurring Natives Under Different Soil Nitrogen Forms and Concentrations

**DOI:** 10.3390/plants14050640

**Published:** 2025-02-20

**Authors:** Jian-Kun Sun, Ming-Chao Liu, Ji-Xin Chen, Bo Qu, Ying Gao, Lin Geng, Li Zheng, Yu-Long Feng

**Affiliations:** 1 College of Ecology and Environment, Southwest Forestry University, Kunming 650224, China; 13998311092@163.com; 2 College of Bioscience and Biotechnology, Shenyang Agricultural University, Shenyang 110866, China; lmc8866@syau.edu.cn (M.-C.L.); 2019200046@syau.edu.cn (J.-X.C.); syau_qb@163.com (B.Q.); 3 Yixian Water Conservancy Affairs Service Center, Jinzhou 121100, China; 18840124411@163.com (Y.G.); 13188121099@163.com (L.G.)

**Keywords:** ammonium, invasive plants, 15N labeling, nitrate, nitrogen addition, nitrogen form acquisition strategies, plasticity, preference

## Abstract

High nitrogen (N) uptake is one of the main reasons for invasive alien plant invasions. However, little effort has been made to compare the effects of different N forms on N uptake between invasive and native plants, especially those on N form acquisition strategies (preference and plasticity), which influence N uptake, and thus exotic plant invasions. Related studies are particularly few in barren habitats, where the effects of N deposition on invasiveness are considered to be much weaker than in fertile habitats. In this study, we grew *Solanum rostratum*, a noxious invader in barren habitats, and the native plants *Leymus chinensis* and *Agropyron cristatum* in both mono- and mixed cultures under nitrate and ammonium addition treatments, and analyzed the effects of the soil N availability and forms on the growth, N uptake, and N form acquisition strategies for these plants. The invader outperformed the natives in N uptake (in most cases) and growth (always) in both mono- and mixed cultures under all N treatments. N addition increased the N uptake and growth of the invader. The advantages of the invader over the natives were higher under ammonium relative to nitrate addition. The growth advantage of the invader was associated with its higher N uptake and higher N-use efficiency. Higher plasticity in N form uptake may contribute to the higher N uptake for the invader when grown in mixed cultures. Our findings indicate that N deposition, particularly in the form of ammonium, may accelerate exotic plant invasions in barren habitats.

## 1. Introduction

Invasive alien plant invasion has emerged as a serious global issue, posing significant threats to environmental security, economic development, and human health [1,2,3]. Invasive plants disrupt nutrient biogeochemical cycling, inducing alterations in native habitats [4,5,6], and threaten the existence of indigenous plants, leading to detrimental effects on biodiversity and biosecurity [7,8,9,10,11]. Understanding the mechanisms underlying invasive alien plant invasions is crucial for the effective risk assessment and control of introduced plants [3,12]. The competition for soil nutrients between invasive and native plants can significantly alter species compositions, structures, and functions in invaded communities [13,14,15].

Nitrogen (N), as an important component of proteins and nucleic acids, plays a pivotal role in plant growth and reproduction [2,16]. A growing body of evidence suggests that in introduced ranges, invasive plants (e.g., *Solidago canadensis* and *Spartina alterniflora*) often exhibit higher N uptake abilities and accumulate higher tissue N contents than their co-occurring natives [15,17,18,19]. This may arise from evolutionary adaptations in their native habitats, where the long-term soil N dynamics and interspecific competition shaped their N acquisition strategies [19,20]. After introduction to novel environments, certain invasive species may capitalize on pre-existing traits such as flexible N acquisition strategies and upregulated gene expressions of N transporters to exploit soil N more effectively than their co-occurring natives [2,19]. Ammonium (NH_4_^+^) and nitrate (NO_3_^−^), the two main available N forms for plants, exhibit significant spatiotemporal variability in soils [21,22]. During the long-term evolutionary process, plants have developed different strategies to utilize these N forms: some plants consistently show a preference for a specific N form regardless of its content and proportion in soils, while others exhibit plasticity by adjusting their N form utilization according to the change in dominant N form in the soil [3,23,24,25,26]. Multiple factors, such as the contents and proportions of different N forms, influence plant N form acquisition strategies. Atmospheric N deposition may also influence N form utilization strategies for both invasive and native plants, as it not only increases soil N availability but also changes N forms in the soil [3].

Divergent N form acquisition strategies profoundly influence plant performance in spatiotemporally heterogeneous habitats characterized by varying N forms [19,26,27]. Plants that utilize the predominant form of soil N may outperform those relying on subdominant N forms [19]. Invasive plants may benefit from either preferential use of the predominant N form or higher plasticity in N form uptake. Such N uptake strategies may provide invasive plants with a competitive advantage over natives, contributing to their invasions. The disparities in N form acquisition strategies between invasive and co-occurring native plants may influence the expressions of invasiveness in invasive plants. Invasive plants may have a higher ability to adjust their N-acquisition strategies to meet their N needs than their co-occurring natives [19,28,29]. However, these differences remain poorly understood.

*Solanum rostratum* Dunal (Solanaceae) is a noxious annual invasive weed that poses considerable ecological and economic challenges in various regions of China, including northeast China, North China, Inner Mongolia, and Xinjiang [30]. It is native to North America, and was first found in 1981 in Chaoyang, Liaoning Province, northeast China. It mainly invades arid and barren habitats, especially disturbed grasslands, roadsides, and field margins [30]. The perennial grasses *Leymus chinensis* (Trin. Ex Bunge) Tzvelev and *Agropyron cristatum* (L.) Gaertn. are the dominant or common species in the steppes of northeast China, Inner Mongolia, and Xinjiang, which can be invaded by *S. rostratum* [3]. The effects of N deposition on the invasiveness of exotic plants such as *S. rostratum*, which invade barren habitats, remain unclear. However, many studies showed that N addition facilitates the invasion success of exotic plants in fertile habitats [31,32]. To address the effects of N deposition on the invasiveness of the invader in terms of both increase in soil N availability and change in soil N forms, we grew the invasive and native plants in fields in both mono- and mixed cultures under nitrate and ammonium addition treatments and measured plant growth, N uptake, and N form acquisition strategies. We hypothesize that (1) *S. rostratum* grows better than the two natives, which is associated with its higher N uptake, use, and efficiency; (2) N addition promotes growth in both the invasive and native plants, with a more pronounced effect on the invader; and (3) the invader uses the dominant N form in the soil, while the natives use the subordinate N form, and both preference and plasticity contribute to these differences.

## 2. Results

### 2.1. Aboveground Biomass

**Figure 1:**
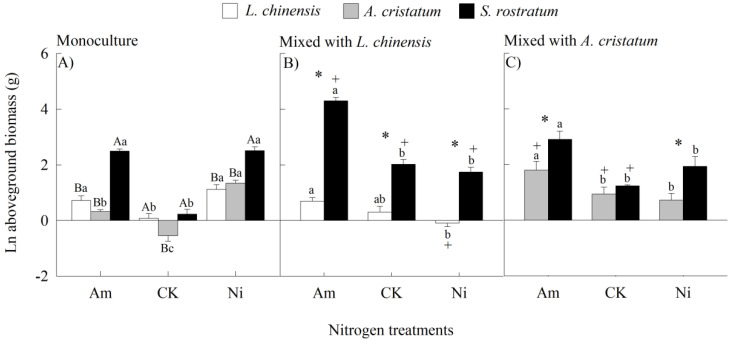
Aboveground biomass of *Leymus chinensis* (open bars), *Agropyron cristatum* (grey bars), and *Solanum rostratum* (closed bars) grown in mono- (**A**) and mixed (**B**,**C**) cultures under different nitrogen treatments. Am, ammonium; CK, control; Ni, nitrate. Raw data were ln-transformed. Mean ± SE (*n* = 4). Different uppercase letters and * represent significant differences between species under same nitrogen treatment in mono- (*p* < 0.05; one-way ANOVA) and mixed (*p* < 0.05, independent sample *t*-test) cultures, respectively. Different lowercase letters represent significant differences among nitrogen treatments for same species under same planting method (*p* < 0.05; one-way ANOVA). + represents significant differences between mono- and mixed cultures for same species under same nitrogen treatment (*p* < 0.05, independent sample *t*-test).

When grown under the control, the difference in aboveground biomass was not significant between the *S. rostratum* and *L. chinensis* grown in monocultures, nor between the *S. rostratum* and *A. cristatum* grown in mixed cultures (Figure 1). In other conditions, however, the invader showed a significantly higher aboveground biomass than the natives. N addition increased the difference in aboveground biomass between the invasive and native species grown in both the mono- and mixed cultures, and ammonium was more effective than nitrate except for the *S. rostratum* and *A. cristatum* in mixed cultures. Consistently, our four-way ANOVA also showed that species and its interactions with N levels and planting methods significantly influenced the aboveground biomass (Table S1).

When grown in monocultures, N addition with either ammonium or nitrate significantly enhanced the aboveground biomass in all three species (Figure 1A). When grown in mixed cultures, however, nitrate addition did not affect biomass in any species, while ammonium addition increased the biomass for *S. rostratum* and *A. cristatum*, but not for *L. chinensis* (Figure 1B,C). When grown in monocultures, the plastic responses to nitrate versus ammonium addition were similar for the invader, whereas the natives responded more greatly to nitrate relative to ammonium addition (Figure 1A). When grown in mixed cultures, all three species responded more greatly to ammonium relative to nitrate-N addition (Figure 1B,C).

When grown under the ammonium addition and control, the aboveground biomass was significantly higher in mixed cultures than in monocultures for the invader and *A. cristatum*, but not for *L. chinensis*. When grown under nitrate addition, a higher biomass was not found in mixed relative to monocultures for any species. In contrast, both *S. rostratum* and *L. chinensis* showed lower biomass in mixed cultures than those in monocultures (Figure 1).

### 2.2. Nitrogen Uptakes

**Figure 2:**
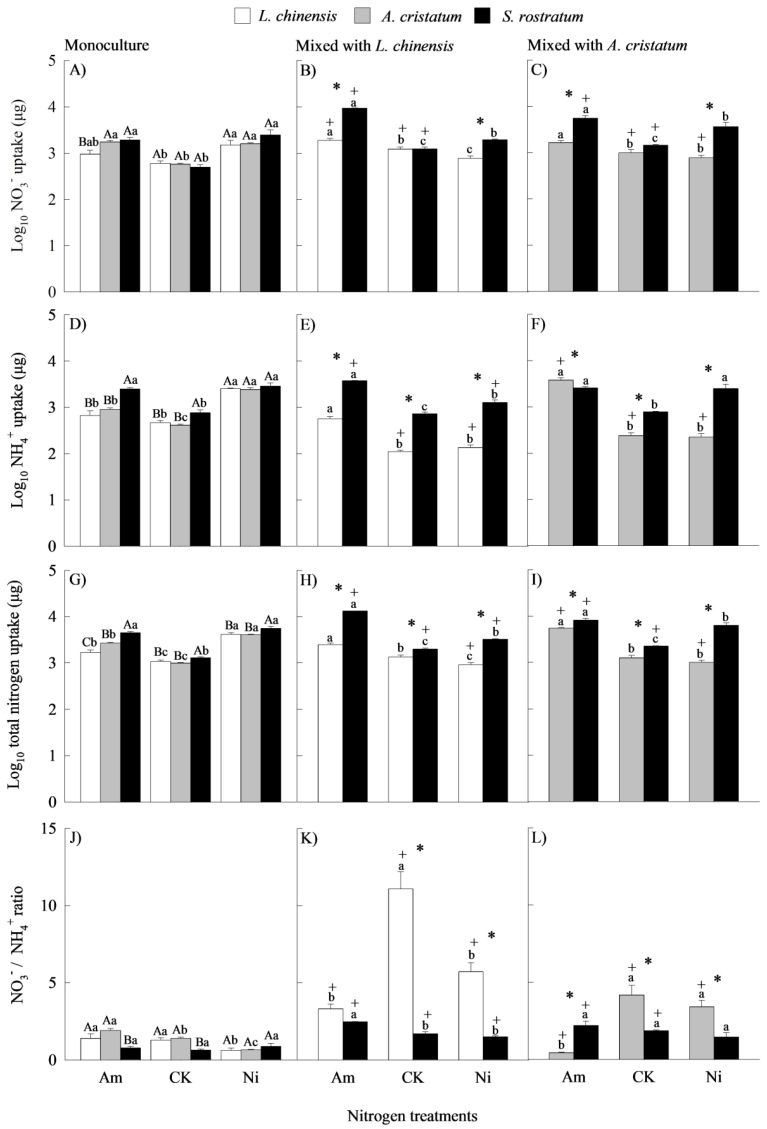
Uptakes of soil NO_3_^−^ (**A**–**C**), NH_4_^+^ (**D**–**F**), and total dissolved inorganic nitrogen (**G**–**I**) and ratio of NO_3_^−^ to NH_4_^+^ (**J**–**L**) in *Leymus chinensis* (open bars), *Agropyron cristatum* (grey bars), and *Solanum rostratum* (closed bars) grown in mono- and mixed cultures under different nitrogen treatments. Am, ammonium; CK, control; Ni, nitrate. Raw data were log10-transformed except for NO_3_^−^/NH_4_^+^ ratio. Mean ± SE (*n* = 3). Different uppercase letters and * represent significant differences between species under same nitrogen treatment in mono- (*p* < 0.05; one-way ANOVA) and mixed (*p* < 0.05, independent sample *t*-test) cultures, respectively. Different lowercase letters represent significant differences among nitrogen treatments for same species under same planting method (*p* < 0.05; one-way ANOVA). + represents significant difference between mono- and mixed cultures for same species under same nitrogen treatment (*p* < 0.05, independent sample *t*-test).

When grown in monocultures, *S. rostratum* displayed significantly higher nitrate-N uptakes than *L. chinensis* under ammonium-N; under other N treatments, however, there were no significant differences in nitrate-N uptakes between the invasive and native species (Figure 2A). When grown in mixed cultures, the nitrate-N uptake was significantly higher for the invasive relative to the native species under both ammonium and nitrate addition treatments, but not under the control (Figure 2B,C). The invader exhibited significantly higher ammonium-N and total inorganic N uptakes than the natives in most cases (Figure 2D–I). Similarly, the invader had higher uptake rates of nitrate-N, ammonium-N, and total inorganic N than the natives in all cases except for *S. rostratum* mixed with *L. chinensis* under ammonium-N treatment (Figure S1A–I).

Our four-way ANOVA showed that N level and its interaction with species significantly affected N uptake (Table S2). N addition increased the uptakes of nitrate-N, ammonium-N, and total inorganic N for the invader grown in both mono- and mixed cultures (Figure 2A–I). N addition in monocultures (except for *L. chinensis* under ammonium-N treatment) and ammonium-N addition in mixed cultures increased the uptakes of nitrate-N, ammonium-N, and total inorganic N for the natives. Nitrate-N addition in mixed cultures, however, did not enhance the N uptakes for the natives (Figure 2A–I). When grown in monocultures, N forms did not significantly affect the uptakes of nitrate-N, ammonium-N, and total inorganic N by the invader (Figure 2A,D,G). For the natives, however, the uptakes of ammonium-N and total inorganic N (but not nitrate-N) were significantly lower under ammonium relative to nitrate-N. When grown in mixed cultures, the uptakes of nitrate-N, ammonium-N (except for the invader mixed with *A. cristatum*), and total inorganic N were significantly higher under ammonium relative to nitrate-N for all three species (Figure 2B,C,E,F,H,I).

Our four-way ANOVA showed that planting method and its interaction with species significantly influenced N uptake (Table S2). When grown in mixed (with two natives) relative to monocultures, the uptakes of nitrate-N, ammonium-N, and total inorganic N were significantly higher in most cases for the invader under the ammonium and control treatments, but not under nitrate treatment (or even lower) (Figure 2A–I). However, similar patterns were not found for the natives (lower in 7 cases, similar in 6 cases, and higher in 5 cases). For total inorganic N, which is more important than either ammonium or nitrate in determining plant performance, the planting methods did not influence the uptake in *L. chinensis* under the ammonium and control treatments, or in *A. cristatum* under the control. When grown in mixed cultures compared with monocultures, the uptake of total inorganic N was lower for the two natives under nitrate-N, but higher only for *L. chinensis* under ammonium-N.

The species, N level, and planting method all significantly influenced the uptake ratios of nitrate-N to ammonium-N based on our four-way ANOVA (Table S2). When grown in monocultures, the invader relative to the natives exhibited significantly lower uptake ratios of nitrate-N to ammonium-N under the ammonium and control treatments, but not under nitrate-N conditions (Figure 2J). When grown in mixed cultures, the invader also showed lower uptake ratios of nitrate-N to ammonium-N under the control and nitrate addition treatments, with similar (mixed with *L. chinensis*) or even higher (mixed with *A. cristatum*) uptake ratios of nitrate-N to ammonium-N under ammonium addition treatment (Figure 2K,L). The N treatments did not significantly influence the uptake ratios of nitrate-N to ammonium-N for the invader grown in either mixed or monocultures except under ammonium treatment mixed with *L. chinensis*, under which the ratio was higher than those under the control and nitrate addition treatments (Figure 2J–L). For *L. chinensis*, N addition reduced the uptake ratios of nitrate-N to ammonium-N grown under all planting and N treatments except under ammonium treatment in monocultures, under which the ratio was similar with that under the control. For the *A. cristatum* grown in monocultures, the uptake ratio of nitrate-N to ammonium-N was lower under nitrate treatment than those under CK and ammonium treatment, while grown in mixed cultures, the ratio was lower under ammonium treatment than those under CK and nitrate treatment. When grown in mixed cultures compared with monocultures, the uptake ratios of nitrate-N to ammonium-N were significantly higher under all N treatments except for *A. cristatum* under ammonium (lower) and *S. rostratum* mixed with *A. cristatum* under nitrate (similar).

### 2.3. Nitrogen Form Preference

**Figure 3:**
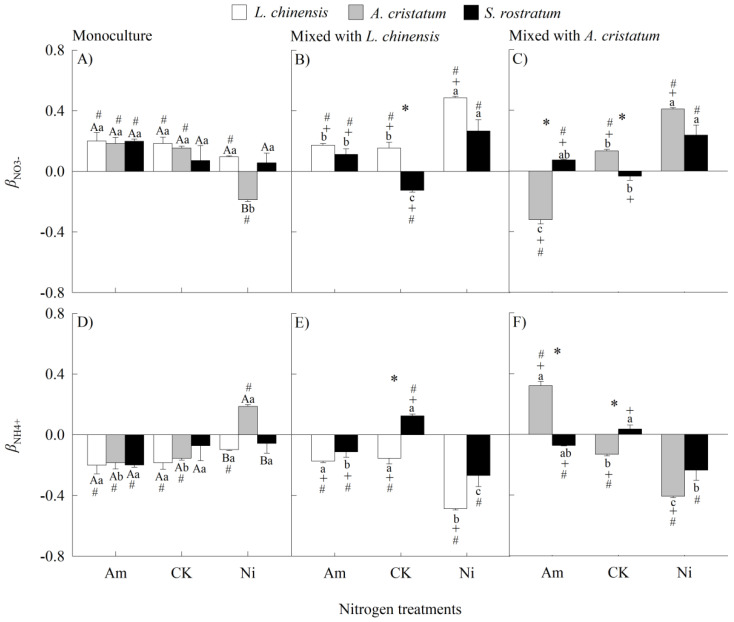
Preference for NO_3_^−^ (**A**–**C**; *β*_NO3_^−^) and NH_4_^+^ (**D**–**F**; *β*_NH4_^+^) in *Leymus chinensis* (open bars), *Agropyron cristatum* (grey bars), and *Solanum rostratum* (closed bars) grown in mono- and mixed cultures under different nitrogen treatments. Am, ammonium; CK, control; Ni, nitrate. Mean ± SE (*n* = 3). Different uppercase letters and * represent significant differences between species under same nitrogen treatment in mono- (*p* < 0.05; one-way ANOVA) and mixed (*p* < 0.05, independent sample *t*-test) cultures, respectively. Different lowercase letters represent significant differences among nitrogen treatments for same species under same planting method (*p* < 0.05; one-way ANOVA). + represents significant difference between mono- and mixed cultures for same species under same nitrogen treatment (*p* < 0.05, independent sample *t*-test). # represents significant difference with 0 (*p* < 0.05, independent sample *t*-test).

The preferences for both NO_3_^−^ (*β*_NO3_^−^) and NH_4_^+^ (*β*_NH4_^+^) were significant (the values of *β*_NO3_^−^ and *β*_NH4_^+^ different with zero) in all three species grown in both mixed and monocultures under all N treatments, except for the invader grown in monocultures under CK and nitrate treatment and mixed with *A. cristatum* under CK treatment (Figure 3). Species, N forms, and N levels all significantly influenced N form preferences according to our four-way ANOVA (Table S3). When grown in monocultures, *A. cristatum* preferred ammonium, while the other two species preferred nitrate under nitrate addition treatment, and under other N treatments, the interspecific differences in N form preferences were not significantly different (Figure 3A,D). When grown in mixed cultures, the invader preferred ammonium and the two natives preferred nitrate under CK; under ammonium addition treatment, the invader preferred nitrate and *A. cristatum* preferred ammonium; and under other N treatments, the interspecific differences in N form preferences were not significantly different (Figure 3B,C,E,F).

When grown in monocultures, N addition did not significantly influence N form preferences except for *A. cristatum* under nitrate addition treatment, under which its preference changed from nitrate (CK) to ammonium (Figure 3A,D). When grown in mixed cultures, nitrate addition increased the preferences for nitrate in all three species; ammonium addition changed the preferences of the invader from ammonium (or no preference) to nitrate, while it did not influence influence the preference of *L. chinensis*, and even decreased *A. cristatum*’s nitrate preference (from nitrate to ammonium) (Figure 3B,C,E,F).

When grown in mixed cultures compared with monocultures, the values of *β*_NO3_^−^ were decreased significantly for all three species under CK (the invader’s preference changed from no preference to ammonium) and ammonium addition treatment (*A. cristatum*’s preference changed from nitrate to ammonium), while the values of *β*_NO3_^−^ were increased for all species (the invader’s preference changed from ammonium or no preference to nitrate) (Figure 3).

### 2.4. Percentage Similarity

**Figure 4:**
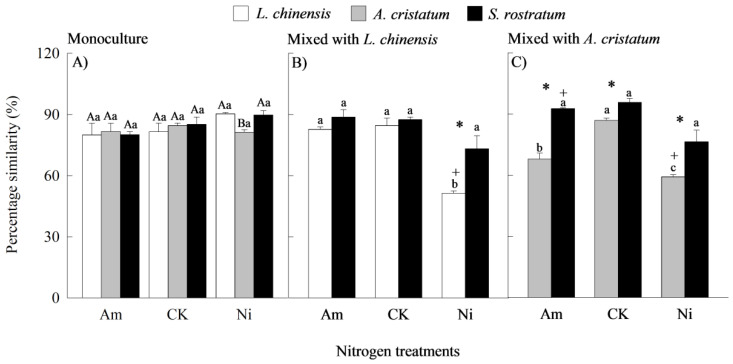
Percentage similarities between plant uptake patterns of different nitrogen forms and their availability patterns in soil for *Leymus chinensis* (open bars), *Agropyron cristatum* (grey bars), and *Solanum rostratum* (closed bars) grown in mono- (**A**) and mixed (**B**,**C**) cultures under different nitrogen treatments. Am, ammonium; CK, control; Ni, nitrate. Mean ± SE (*n* = 3). Different uppercase letters and * represent significant differences between species under same nitrogen treatment in mono- (*p* < 0.05; one-way ANOVA) and mixed (*p* < 0.05, independent sample *t*-test) cultures, respectively. Different lowercase letters represent significant differences among nitrogen treatments for same species under same planting method (*p* < 0.05; one-way ANOVA). + represents significant difference between mono- and mixed cultures for same species under same nitrogen treatment (*p* < 0.05, independent sample *t*-test).

Species, N forms and levels, and planting methods all significantly influenced the percentage similarities between the plant uptake patterns of different N forms and their availability patterns in the rhizosphere soil (Table S4). When grown in monocultures, the percentage similarities did not significantly differ among the invasive and native species except for *A. cristatum* under nitrate addition treatment, under which the values of its percentage similarity were lower than those of the two other species (Figure 4A). When grown in mixed cultures, the values of the percentage similarity were higher for the invader than for the natives under all N treatments, but the differences did not reach significant levels in two cases (the invader competed with *L. chinensis* under CK and ammonium treatment) (Figure 4B,C).

N addition did not significantly influence the percentage similarities for all species grown in monocultures under all N treatments (Figure 4A). When grown in mixed culture, N addition decreased the values of the percentage similarity for *L. chinensis* under nitrate treatment, and those for *A. cristatum* under both ammonium and nitrate addition treatments, while it did not significantly influence the percentage similarity for the invader (Figure 4B,C).

When grown in mixed cultures, the values of the percentage similarity were lower for *L. chinensis* and *A. cristatum* under nitrate treatment, but higher for *S. rostratum* mixed with *A. cristatum* under ammonium treatment than those in monocultures (Figure 4).

### 2.5. Correlation Between Biomass and N Uptakes and That Between N Preference and Uptake Ratios of Nitrate-N to Ammonium-N

**Figure 5:**
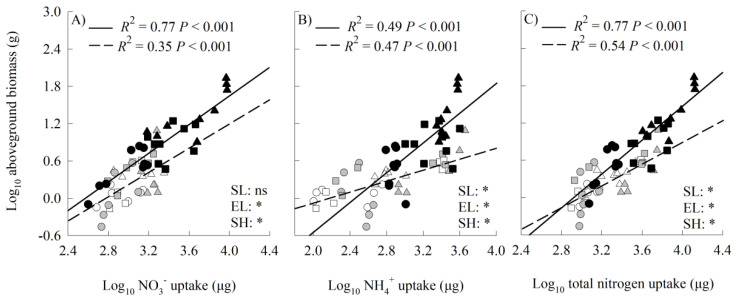
Standardized major axis regressions between aboveground biomass and nitrogen uptakes of NO_3_^−^ (**A**), NH_4_^+^ (**B**), and total dissolved inorganic nitrogen (**C**) for invasive (solid line) and native (dashed line) species grown in both mono- and mixed cultures under different nitrogen treatments. Open symbols, *Leymus chinensis*; grey symbols, *Agropyron cristatum*; closed symbols, *Solanum rostratum.* Circles, control; triangles, ammonium; squares, nitrate. SL, slope; EL, intercept; SH, shift along common slope. *, significant differences (*p* < 0.05); ns, non-significant differences.

The aboveground biomass increased positively with increasing uptakes of nitrate-N, ammonium-N, and total inorganic N for both the invasive and two native species (Figure 5). The intercepts of the correlations between biomass and nitrate-N uptake were significantly different between the invader and the two natives, with the invader being higher (Figure 5A). Compared with the two natives, the invader was distributed at the end, with higher values of nitrate-N uptakes along the common slope. For the correlations between biomass and uptakes of ammonium-N and total inorganic N, the values of the slope, intercept, and shift were also significantly different between the invader and the two natives (Figure 5B,C). The invader was distributed at the higher end of the uptakes of ammonium-N and total inorganic N, and had a higher biomass at the same values of N uptakes when the N uptakes were relatively high.

**Figure 6:**
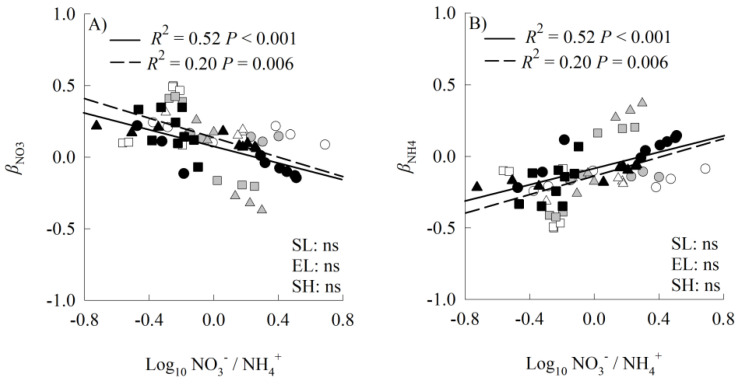
Standardized major axis regressions between *β*_NO3_^−^ (**A**) or *β*_NH4_^+^ (**B**) and NO_3_^−^/NH_4_^+^ ratio for invasive (solid line) and native (dashed line) species grown in both mono- and mixed cultures under different nitrogen treatments. Open symbols, *Leymus chinensis*; grey symbols, *Agropyron cristatum*; closed symbols, *Solanum rostratum.* Circles, control; triangles, ammonium; squares, nitrate. SL, slope; EL, intercept; SH, shift along common slope. ns, non-significant differences (*p* < 0.05).

The values of *β*_NO3_^−^ decreased significantly with increasing values of the nitrate-N to ammonium-N ratios for both the invader and the two natives, while the values of *β*_NH4_^+^ increased (Figure 6).

## 3. Discussion

In line with our hypothesis, the invasive plant *S. rostratum* demonstrated superior growth compared with its co-occurring natives *L. chinensis* and *A. cristatum* grown in both mixed and monocultures, which was increased by N (either nitrate and ammonium) addition. Numerous studies found that increasing soil N availability promotes the growth advantage of invasive plants over natives, thereby facilitating their invasions [31,32,33]. For the two natives, N addition significantly increased growth when grown in monocultures, but not in most cases when grown in mixed cultures, which was not true for the invader (increased in both mixed and monocultures). These findings indicate that planting methods may alter the effects of N on plant growth [34,35]. The relatively low responses of the natives to N addition in mixed cultures may be associated with the higher N uptake of the invader, which leads to a substantial proportion of the supplemented N being absorbed by the invader. N addition increased the total leaf area for the invader, potentially shading the natives, which may be another reason for the low responses to N addition in the natives [36,37]. The roles of soil N availability in plant invasion dynamics have been studied extensively [33]. Our findings further show the roles of soil N forms in the successful invasions of introduced plants. The growth advantage of *S. rostratum* over its co-occurring natives was greater in ammonium relative to nitrate-N addition treatment. This N-form-driven effect highlights the need to incorporate soil N forms into invasion prediction models, which currently rely solely on total N metrics. In addition, the targeted manipulation of soil N forms may have the potential to control invasive plants.

A few studies found that increasing soil N availability facilitates the invasion of introduced species with conservative resource-use strategies such as *S. rostratum* [38], which has been demonstrated in many introduced species with acquisitive resource-use strategies [39,40,41]. The invader generally invades barren habitats with low precipitation and soil nutrients [30], and its plastic response to nutrient addition is lower than its native congener [38]. However, Sun et al. (2023) reported that the growth of the invader did not increase significantly under the same level of N addition (6 g N m^−2^) [3], inconsistent with the current study. This discrepancy may stem from the differences in soil fertility (Table S5). Based on the soil fertility assessment criteria [42], soil nutrients were high in the study conducted by Sun et al. (2023) [3], but low in the current study. The soil nutrient level may affect plant response to N addition [43,44]. For instance, Xu et al. (2014) found that N addition increases plant yield more significantly in nutrient-deprived soils than in nutrient-abundant soils [43]. These results highlight the importance of considering soil nutrient availability and the nutrient-use strategies of studied species when studying the effects of N deposition on the invasiveness of introduced plants.

Consistent with our hypothesis, the uptakes of nitrate-N, ammonium-N, and total inorganic N were higher for the invasive relative to the two natives in most cases, providing a possible explanation for the growth advantage of the invader. The aboveground biomass positively correlated with the uptakes of these N forms for all species. Similar patterns were also observed in other invasive plants [15,19]. A higher N uptake rate contributed to the higher N uptake of the invader. The invader may also have more absorptive roots than the natives, which can help plants to absorb N [19], as the invader absorbed more N than the natives even if the inter-group difference in N uptake rates was not significant. In addition, the invader may have a higher N-use efficiency than the two natives, which may also contribute to its growth advantage [15,45,46]. Based on our SMA, biomass was higher for the invasive relative to the native species at a given nitrate-N uptake, and the slopes of the correlations between the biomass and the uptakes of ammonium-N and total inorganic N were also higher for the invader, indicating higher N-use efficiency for the invader. These results indicate that the invader may allocate more N absorbed to growth than the two natives, especially when N uptake is high. Feng et al. (2009, 2011) found that the invasive plant *Ageratina adenophora* allocates more leaf N to photosynthesis, enhancing its resources-use efficiency and invasiveness [45,46].

All three species preferred nitrate in most cases, as indicated by our ^15^N labeling experiment. However, this preference was inconsistent with the results from our growth experiment. For example, the invader responded similarly to either nitrate or ammonium addition when grown in monocultures (no preference); when grown in mixed cultures, however, the invader as well as the two natives responded more greatly to ammonium relative to nitrate addition (preference for ammonium). These discrepancies suggest that isotopic labeling experiments are essential for accurately determining plant N form preference. Our study demonstrated that species, N levels, N forms, planting methods, and their interactions affected N form preference in complex ways. Interestingly, the less of a given N form in the rhizosphere soils, the more the three plants preferred that N form. Similar results were found by Guan et al. (2023) when they compared the invasive plant *Solidago canadensis* and its co-occurring native plant *Artemisia vulgaris* in three habitats from four locations [19]. These results seem to be inconsistent with the results that the three species were more likely to prefer the N form artificially added to the soils when grown in mixed cultures. However, the proportional content of the N form added may not always increase in rhizosphere soils. In contrast, the content decreased for the studied species in most cases when grown in mixed cultures.

Consistent with our hypothesis, when grown in mixed cultures, the invader preferentially utilized the dominant N form in the soil under ammonium addition, while *A. cristatum* preferred the subordinate N form (Figure S2). Such strategies enable the invader to acquire more N, therefore facilitating its growth and invasion [19]. When grown in mixed cultures, the higher plasticity in N form uptake for the invader relative to the two natives may help it quickly adapt to changes in soil N forms, contributing to its higher N uptake, and thus to its higher aboveground biomass. Until now, only a few studies estimated plasticity in N form uptake [19,21,47], and thus its effects on the invasiveness of introduced plants need further study.

## 4. Materials and Methods

### 4.1. Study Sites and Species

The study was conducted in Daling River National Wetland Park, Yixian County, Liaoning Province, China (41°34′19″ N, 121°9′9″ E; 70 m asl). Characterized by a continental monsoon climate, this region experiences a mean annual temperature of 8.0 °C and a mean annual precipitation of 600 mm [48]. In our experimental field, the soil composition was predominantly sandy, with carbon and total N contents of 4470 and 340 μg g^−1^ dry weight (dw) soil, respectively. The soil contents of available phosphorus and potassium, ammonium-N, and nitrate-N were 8.17, 68.43, 22.63, and 5.08 μg g^−1^ dw soil, respectively, and the soil pH was 8.17. These results indicate that the soil was poor according to the soil nutrient evaluation criteria [42]. Dominant plant species in this region include *L. chinensis*, *A*. *cristatum*, and *Digitaria sanguinalis*.

The invasive plant *S*. *rostratum* and its two co-occurring native plants, *L*. *chinensis* and *A*. *cristatum*, were compared in this study. *S*. *rostratum*, an annual herb in Solanaceae, is native to North America, first recorded in 1981 in Chaoyang, Liaoning Province, China, but has emerged as one of the most pernicious invasive weeds in northeast China, North China, Inner Mongolia, and Xinjiang. It mainly invades arid and barren habitats, especially disturbed grasslands, roadsides, and field margins [30]. *L*. *chinensis* and *A*. *cristatum* are perennial grasses (Poaceae), and common or dominant species in the arid or semi-arid grasslands of northeast China and Inner Mongolia, which is often invaded by *S*. *rostratum* [3].

The seeds of *S*. *rostratum* were collected in October 2021 in Baicheng, China. The seeds of *L*. *chinensis* and *A*. *cristatum* were bought from Shenyang Jinfuyou Seed Co., Ltd. (Shenyang, China).

### 4.2. Plant Cultures and N Treatments

In May 2022, the experimental field was flattened, and all vegetations (including rhizomes) were removed. The field was then tilled to a depth of 30 cm, and 1.2 m × 1.2 m plots were established with an interspace of 1.2 m. The seeds of the three species were stratified in moist sand at 4 °C for 14 d, sterilized using a 0.5% KMnO_4_ for 30 min, rinsed thoroughly with distilled water, and then sowed in each plot at a depth of 0.5 cm, with 20 seeds per site. The seedlings were grown in both mono- and mixed cultures. For the monocultures, each plot contained 81 sowing sites of each species, arranged in a 9 × 9 grid [3]. For the mixed cultures, each plot contained 16 sowing sites of *S*. *rostratum* and 65 sowing sites of either *L*. *chinensis* or *A*. *cristatum*, arranged in a 9 × 9 grid with interspecific planting [3]. When the seedlings grew to ≈10 cm tall, one plant was retained (others were pulled out) per site for *S*. *rostratum* versus five plants for both *L*. *chinensis* and *A*. *cristatum*. The planting density for the two natives in the monocultures (≈281 plants m^−2^) was based on the results of our field investigation and those of Ping et al. (2007) [49].

Three N treatments were applied one week after thinning: the control (CK; 0 g N m^−2^; H_2_O), ammonium-N (Am; 6 g N m^−2^; using NH_4_Cl solution), and nitrate-N (Ni; 6 g N m^−2^; NaNO_3_). The amount of N applied was based on the atmospheric N deposition in China [50]. The N was applied six times, with a 5-day interval (1 g N m^−2^ once). Nitrification inhibitor (dicyandiamide; 9.75 g m^−2^) was applied into all plots to inhibit the transformation of ammonium-N to nitrate-N [51]. In total, we had 60 plots [3 N treatments × 5 planting methods (3 monocultures + 2 mixed cultures) × 4 replicates]. The plots were irrigated with droppers (≈5 mm of rainfall every 6 days), and weeds were pulled out when found.

### 4.3. ^15^N Labeling, Sampling, and Measurements

We used ^15^N isotope as a tracer to quantify the plant uptakes of soil N with different forms. This method assumes that the isotope fractionation is negligible during plant N uptake when exposed to a low concentration of labeled N [19].

In early August, three plots were randomly chosen for each culture method and N treatment. For each species in each of the plots, three sowing sites (3 individuals for the invader and/or 15 for the natives) were selected for ^15^N labeling [^15^NH_4_Cl, Na^15^NO_3_, and H_2_O (control)]. Around each site (*r* = 3.0 cm), 30 mL of 1.11 mmol N L^−1^ labeling solution (^15^N% > 99.12%) or distilled water were injected into the soil (10 cm depth), which was based on our preliminary experiments. For the details, please see Guan et al. (2023) [19]. Using this method, the injected solution was dispersed evenly into a soil cylinder with a 6.0 cm radius and 15 cm depth around each site. The N added increased the soil N concentration by 1.0 μg g^−1^ dw soil, which was less than 10% of the soil background N pool, and thus avoided fertilizing effects [52,53].

Aboveground parts (above the soil surface) and the roots in the soil cylinder mentioned above were collected separately for the labeled and control individuals after 48 h of ^15^N labeling [19]. The roots were gently rinsed with tap water and immersed into 0.5 mmol L^−1^ CaCl_2_ solution for 30 min to remove the ^15^N adhered to the root surfaces [54,55]. The roots were further rinsed twice with deionized water, dried with filter papers, and then stored in envelopes. The roots and aboveground parts from the same sampling individual(s) were oven-dried at 60 °C for 48 h and weighed, respectively. Both parts were mixed evenly, ground into a fine powder with a ball mill (GT 200, Grinder, Beijing, China), and used for determining the total N content and ^15^N atom% excess with elemental analysis–isotope ratio mass spectrometry (Flash 2000HT + Delta V Advantage, Thermo, Waltham, Germany). The measurement was conducted by the Third Institute of Oceanography, Ministry of Natural Resources, Xiamen, China.

Rhizosphere soils from the unlabeled individuals were collected for the invasive and native species (3 replicates) using the method described by Zhao et al. (2020) [10]. The soil was sieved through a 2 mm mesh, and NH_4_^+^ and NO_3_^−^ were extracted using 2 mol L^−1^ KCl solution. The concentrations of NH_4_^+^ and NO_3_^−^ in the extraction were measured using an Automated Chemistry Analyzer (AA3, Seal, Hannover, Germany). For the labeled plants, the N contents of a specific form in their rhizosphere soils were calculated as the sum of the contents measured for the unlabeled plants and the content added (1.0 μg g^−1^ dw soil). The ratios of nitrate-N to ammonium-N in the rhizosphere soils were calculated for all plants sampled.

In late August, one (for the invader) or five (for the natives) plants were randomly chosen from each plot, and the aboveground parts were harvested, oven-dried at 60 °C for 48 h, and then weighed.

### 4.4. Calculations

We first calculated the uptakes of the labeled ^15^N from a specific form (^15^NH_4_^+^ or ^15^NO_3_^−^) for the labeled plants using their ^15^N atom% excess (APE), total biomass (the aboveground parts and the roots in the soil cylinders), and N contents. Using the values of the uptakes of the labeled ^15^N and the ratios of the labeled ^15^N to the N naturally existing in the soils, we then calculated the uptakes of the latter (both ^14^N and ^15^N) (actual N uptake) for the labeled plants. The uptake rates for each form of N were calculated for the labeled plants using their uptakes, root biomass, and the duration labeled (48 h). Finally, we calculated the preference and plasticity in N form uptake for the labeled plants. For details, please see Guan et al. (2023) [19].

### 4.5. Statistical Analysis

The effects of species, N forms, N levels, planting methods, and their interactions on the variables were tested using a four-way analysis of variance (ANOVA). A one-way ANOVA was used to test for the difference in each variable among different N treatments for the same species grown in mono- or mixed cultures, and for the difference in each variable among the three plant species grown in monocultures under the same N treatment. An independent sample *t*-test was applied to analyze the difference in each variable between the invasive and native plants grown in mixed cultures under the same N treatment, the difference in plant N form preference compared with 0, and the difference between the mono- and mixed cultures for the same species under the same N treatment. All of the analyses were performed using PASW Statistics 18.0 (SPSS Inc., Chicago, IL, USA). Prior to these analyses, the data were checked for normality and homogeneity, and the data were transformed using ln or log (see figures) if they did not meet the requirements of ANOVA.

Standardized major axis regression (SMA) was used to analyze the relationship between the aboveground biomass and N uptake, and their differences between the invasive and native plants. The relationship between the plant N form preference and soil nitrate-N/ammonium-N ratio and the interspecific differences were also analyzed using SMA. SMA was performed using the “smatr” package in R 3.6.1 (R Development Core Team, Vienna, Austria).

## 5. Conclusions

Our study shows that (1) *S. rostratum* outperforms its co-occurring natives in terms of growth, and its growth advantage may be associated with its higher N uptake and higher N-use efficiency; (2) N addition enhances its growth advantage over the natives, promoting its invasion, and ammonium addition is more conducive to the invasion of the invader than nitrate addition; and (3) a higher plasticity in N form uptake may contribute to the higher N uptake for the invader when grown in mixed cultures. Our results indicate that N deposition, especially ammonium deposition, can also promote the invasion of introduced plants with conservative resource-use strategies in arid and barren habitats. Future studies on nitrogen deposition effects should consider soil nutrient availability and plant resource-use strategies to better understand the invasion dynamics of introduced species.

## Data Availability

All data generated or analyzed during this study are included in the published article (Supplementary Files) and also available from the corresponding author on reasonable request.

## Supplementary Materials

The following supporting information can be downloaded at https://www.mdpi.com/article/10.3390/plants14050640/s1. Table S1: Effects of species, nitrogen forms, nitrogen levels, planting methods, and their interactions on aboveground biomass; Table S2: Effects of species, nitrogen forms, nitrogen levels, planting methods, and their interactions on uptakes of NO_3_^−^, NH_4_^+^, total dissolved inorganic nitrogen, and ratio of NO_3_^−^ to NH_4_^+^ absorbed by plant; Table S3: Effects of species, nitrogen forms, nitrogen levels, planting methods, and their interactions on preference for nitrate and ammonium; Table S4: Effects of species, nitrogen forms, nitrogen levels, planting methods, and their interactions on percentage similarities between plant uptake patterns of different nitrogen forms and their availability patterns in soil; Table S5: Soil physiochemical properties of study sites; Figure S1: Uptake rates of soil nitrate-N, ammonium-N, and total inorganic N and uptake ratio of nitrate-N to ammonium-N by *Leymus chinensis*, *Agropyron cristatum*, and *Solanum rostratum* grown in mono- and mixed cultures under different nitrogen treatments; Figure S2: Soil inorganic nitrogen contents and NO_3_^−^/NH_4_^+^ ratios in rhizosphere soils of *Leymus Chinensis*, *Agropyron Cristatum*, and *Solanum rostratum* grown in mono- and mixed cultures under different nitrogen treatments.
