# Supplementary material for: Higher Nitrogen Uptakes Contribute to Growth Advantage of Invasive Solanum rostratum over Two Co-Occurring Natives Under Different Soil Nitrogen Forms and Concentrations
Source: Plants (Basel). 2025 Feb 20;14(5):640. doi: 10.3390/plants14050640 (PMC11901527; doi:10.3390/plants14050640)
Supplement: Supplementary file 1 [file plants-14-00640-s001.zip › plants-3422171-supplementary.pdf]

Table S1 Effects of species (*n* = 3; S), nitrogen forms (*n* = 2; NF), nitrogen levels (*n* = 2; NL), planting methods (*n* = 2; PM), and their interactions on aboveground biomass.

	df	*F*-values	*P*-values
S	2	70.0	**<0.001**
NF	1	2.8	0.099
NL	1	102.3	**<0.001**
PM	1	15.4	**<0.001**
S × NF	2	1.3	0.284
S × NL	2	10.5	**<0.001**
S × PM	2	13.1	**<0.001**
NF × NL	1	2.8	0.099
NF × PM	1	17.3	**<0.001**
NL × PM	1	23.2	**<0.001**
S × NF × NL	2	1.3	0.284
S × NF × PM	2	0.4	0.686
S × NL × PM	2	0.4	0.695
NF × NL × PM	1	17.3	**<0.001**
S × NF × NL × PM	2	0.4	0.686

Significant effects are shown in bold ($P < 0.05$).

Table S2 Effects of species (*n* = 3; S), nitrogen forms (*n* = 2; NF), nitrogen levels (*n* = 2; NL), planting methods (*n* = 2; PM), and their interactions on uptakes of NO_3^-, NH_4^+, total dissolved inorganic nitrogen (DIN), and the ratio of NO_3^- to NH_4^+ absorbed by the plant.

		Uptake of nitrogen						Uptake ratio of	
Source of		NO_3^-		NH_4^+		DIN		NO_3^- to NH_4^+	
variation	*df*	*F*-values	*P*-values	*F*-values	*P*-values	*F*-values	*P*-values	*F*-values	*P*-values
S	2	31.0	**<0.001**	241.9	**<0.001**	119.1	**<0.001**	199.1	**<0.001**
NF	1	9.1	**0.004**	14.8	**<0.001**	16.4	**<0.001**	2.8	0.097
NL	1	181.9	**<0.001**	509.2	**<0.001**	474.8	**<0.001**	122.0	**<0.001**
PM	1	58.4	**<0.001**	182.4	**<0.001**	2.2	0.146	563.7	**<0.001**
S × NF	2	0.3	0.750	6.4	**0.003**	4.0	**0.024**	2.2	0.120
S × NL	2	30.3	**<0.001**	3.5	**0.037**	29.8	**<0.001**	61.5	**<0.001**
S × PM	2	17.5	**<0.001**	67.5	**<0.001**	20.1	**<0.001**	165.4	**<0.001**
NF × NL	1	9.01	**0.004**	14.8	**<0.001**	16.4	**<0.001**	2.8	0.097
NF × PM	1	23.9	**<0.001**	139.9	**<0.001**	105.6	**<0.001**	17.6	**<0.001**
NL × PM	1	33.2	**<0.001**	0.3	0.608	37.0	**<0.001**	111.5	**<0.001**
S × NF × NL	2	0.3	0.750	6.4	**0.003**	4.0	**0.024**	2.2	0.120
S × NF × PM	2	0.9	0.429	21.4	**<0.001**	4.3	**0.017**	9.2	**<0.001**
S × NL × PM	2	3.0	0.056	0.3	0.746	4.9	**0.010**	48.0	**<0.001**
NF × NL × PM	1	23.9	**<0.001**	139.9	**<0.001**	105.6	**<0.001**	17.6	**<0.001**
S × NF × NL × PM	2	0.9	0.429	21.4	**<0.001**	4.3	**0.017**	9.2	**<0.001**

Significant effects are shown in bold ($P < 0.05$).

Table S3 Effects of species (n = 3; S), nitrogen forms (n = 2; NF), nitrogen levels (n = 2; NL), planting methods (n = 2; PM), and their interactions on preference for nitrate (β_{NO3^-}) and ammonium (β_{NH4^+}).

Source of variation	*df*	β_{NO3^-} *F*-values	β_{NO3^-} *P*-values	β_{NH4^+} *F*-values	β_{NH4^+} *P*-values
S	2	25.1	**<0.001**	25.1	**<0.001**
NF	1	8.6	**0.005**	8.6	**0.005**
NL	1	4.2	**0.046**	4.2	**0.046**
PM	1	0.4	0.513	0.4	0.513
S × NF	2	2.3	0.105	2.3	0.105
S × NL	2	24.3	**<0.001**	24.3	**<0.001**
S × PM	2	5.3	**0.008**	5.3	**0.008**
NF × NL	1	8.6	**0.005**	8.6	**0.005**
NF × PM	1	82.7	**<0.001**	82.7	**<0.001**
NL × PM	1	22.7	<0.001	22.7	<0.001
S × NF × NL	2	2.3	0.105	2.3	0.105
S × NF × PM	2	13.8	**<0.001**	13.8	**<0.001**
S × NL × PM	2	1.8	0.179	1.8	0.179
NF × NL × PM	1	82.7	**<0.001**	82.7	**<0.001**
S × NF × NL × PM	2	13.8	**<0.001**	13.8	**<0.001**

Significant effects are shown in bold ($P < 0.05$).

Table S4 Effects of species (*n* = 3; S), nitrogen forms (*n* = 2; NF), nitrogen levels (*n* = 2; NL), planting methods (*n* = 2; PM), and their interactions on percentage similarity between plant uptake pattern of different nitrogen forms and their availability pattern in soil.

variation	*df*	*F*-values	*P*-values
S	2	13.5	**<0.001**
NF	1	5.5	**0.023**
NL	1	40.6	**<0.001**
PM	1	11.6	**0.001**
S × NF	2	0.8	0.473
S × NL	2	4.1	**0.022**
S × PM	2	23.9	**<0.001**
NF × NL	1	5.5	**0.023**
NF × PM	1	23.9	**<0.001**
NL × PM	1	41.7	<**0.001**
S × NF × NL	2	0.8	0.473
S × NF × PM	2	3.2	**0.049**
S × NL × PM	2	2.7	**0.078**
NF × NL × PM	1	23.9	**<0.001**
S × NF × NL × PM	2	3.2	**0.049**

Significant effects are shown in bold ($P < 0.05$).

Table S5 Soil physiochemical properties of the study sites.

Study sites	Total N (mg g^{-1} dw soil)	Total C (mg g^{-1} dw soil)	C : N ratio	Organic matter (mg g^{-1} dw soil)	Available potassium (μg g^{-1} dw soil)	Available phosphorus (μg g^{-1} dw soil)	Total dissolved inorganic nitrogen (μg g^{-1} dw soil)
Baicheng [3]	0.83±0.04a	8.21±0.54a	9.96±0.41b	55.71±3.59a	372.77±32.48a	70.43±7.47a	41.65±2.14a
Yixian (This study)	0.34±0.04b	4.47±0.79b	12.72±0.83a	18.12±1.06b	68.43±15.51b	8.17±1.41b	27.70±1.99b

Note: Mean ± SE (n = 12 for Baicheng; n = 6 for Yixian). Different lowercase letters indicate significant differences between study sites ($P < 0.05$; independent samples t-test).

Figure S1 Uptake rates of soil nitrate-N, ammonium-N, and total inorganic N; and uptke ratio of nitrate-N to ammonium-N by *Leymus chinensis* (open bars), *Agropyron cristatum* (grey bars) and *Solanum rostratum* (closed bars) grown in mono- and mixed cultures under different nitrogen treatments. Am, ammonium; CK, control; Ni, nitrate. The raw data were log10-transformed except for NO_3^- / NH_4^+ ratio. Mean ± SE (n = 3). For each replicate, 5 individuals of *L. chinensis* and *A. cristatum*; 1 individual of *S. rostratum*. Different uppercase letters and * represent significant differences between species under the same nitrogen treatment in mono- ($P < 0.05$; one-way ANOVA) and mixed ($P < 0.05$, independent samples *t*-test) cultures, respectively. Different lowercase letters represent significant differences among nitrogen treatments for the same species under the same planting method ($P < 0.05$; one-way ANOVA). + represents a significant difference between the mixed and monocultures for the same species under the same nitrogen treatment ($P < 0.05$, independent samples *t*-test).

Figure S2 Soil inorganic nitrogen contents and NO_3^- / NH_4^+ ratios in rhizosphere soils of *Leymus Chinensis* (open bars), *Agropyron Cristatum* (grey bars) and *Solanum rostratum* (closed bars) grown in mono- and mixed cultures under different nitrogen treatments. Am, ammonium; CK, control; Ni, nitrate. The raw data were log10-transformed except for soil NO_3^- / NH_4^+ ratio. Mean ± SE (n = 3). For each replicate, 5 individuals of *L. chinensis* and *A. cristatum*; 1 individual of *S. rostratum*. Different uppercase letters and * represent significant differences between species under the same nitrogen treatment in mono- ($P < 0.05$; one-way ANOVA) and mixed ($P < 0.05$, independent samples *t*-test) cultures, respectively. Different lowercase letters represent significant differences among nitrogen treatments for the same species under the same planting method ($P < 0.05$; one-way ANOVA). + represents

significant difference between the mixed and monocultures for the same species under the same nitrogen treatment ($P < 0.05$, independent samples *t*-test).

Figure S1

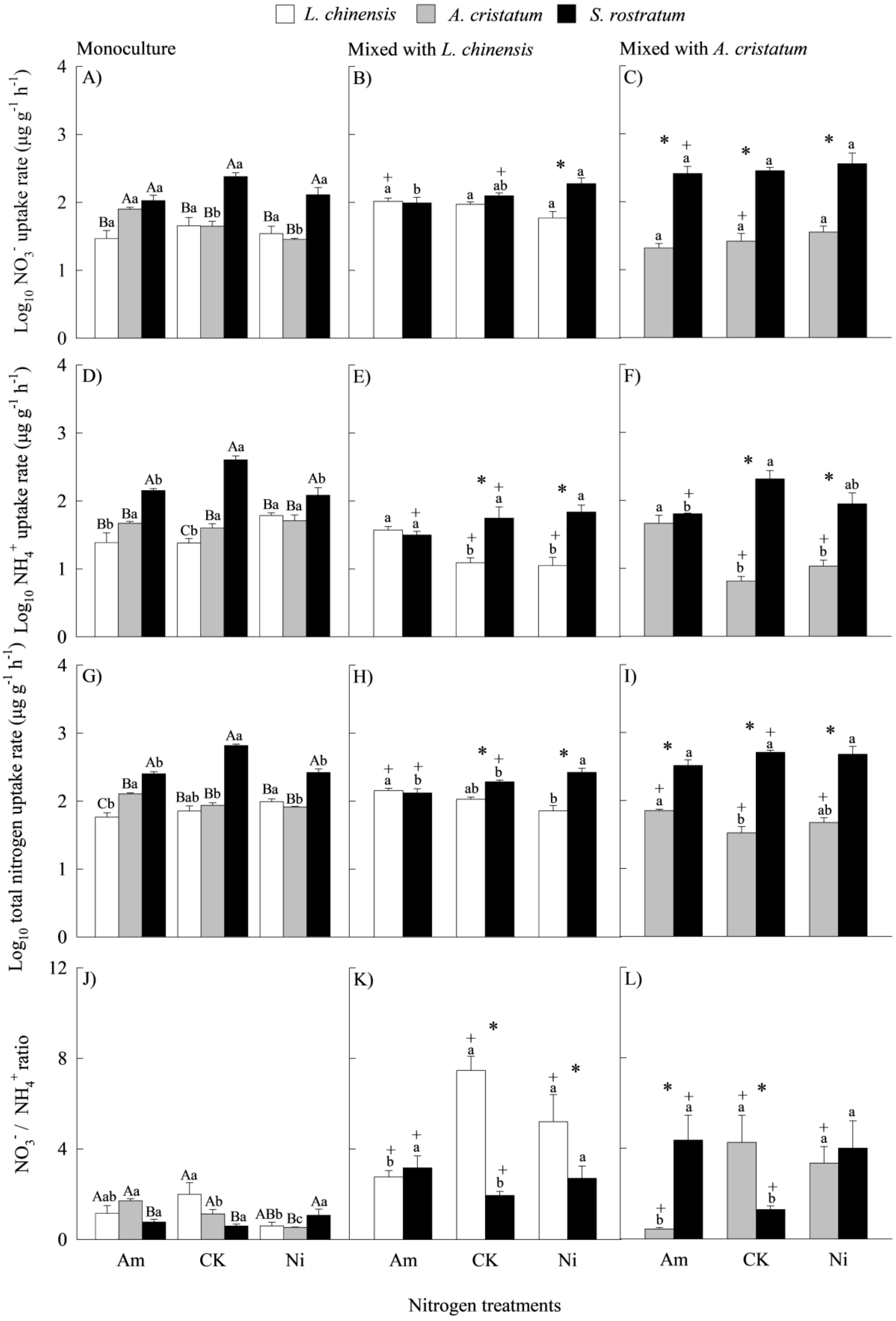

L. chinensis
A. cristatum
S. rostratum
Monoculture
Mixed with L. chinensis
Mixed with A. cristatum
A)
B)
C)
D)
E)
F)
G)
H)
I)
J)
K)
L)
Log10 NO3- uptake rate (μg g-1 h-1)
Log10 NH4+ uptake rate (μg g-1 h-1)
Log10 total nitrogen uptake rate (μg g-1 h-1)
NO3- / NH4+ ratio
Am
CK
Ni
Nitrogen treatments

Figure S2

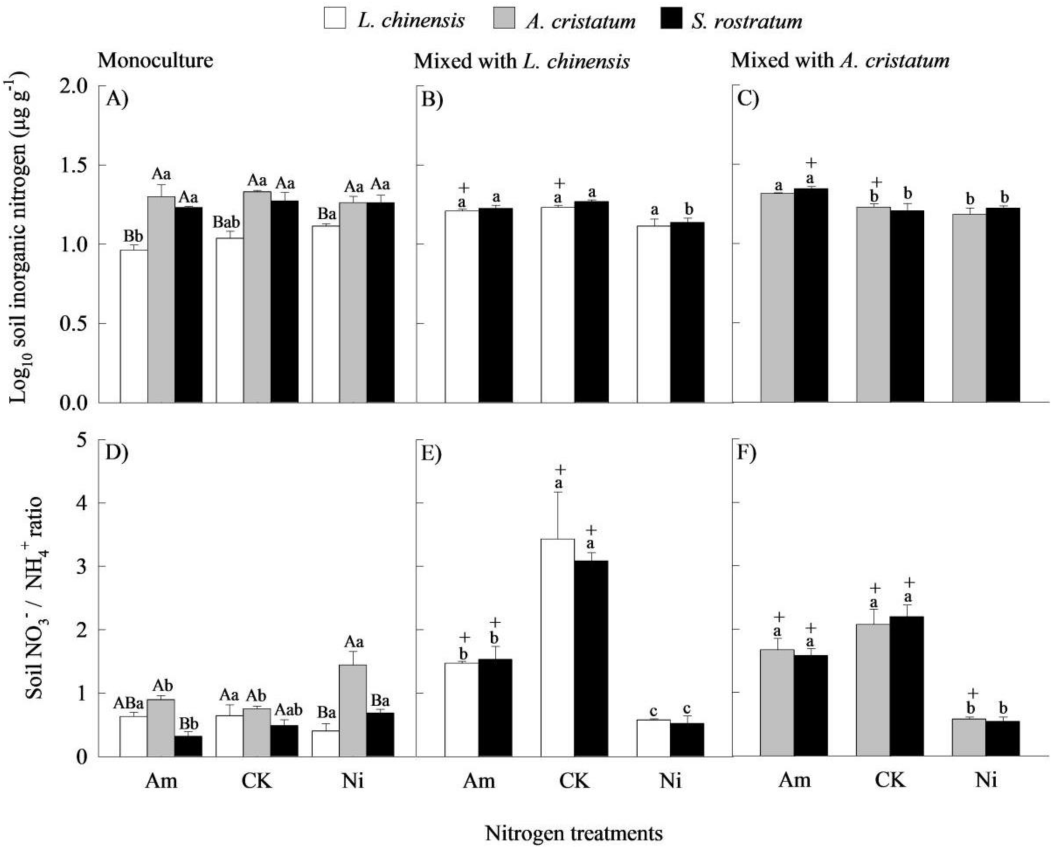

L. chinensis
A. cristatum
S. rostratum
Monoculture
Mixed with L. chinensis
Mixed with A. cristatum
Log10 soil inorganic nitrogen (μg g-1)
A)
B)
C)
Soil NO3- / NH4+ ratio
D)
E)
F)
Am
CK
Ni
Nitrogen treatments
